# Sources of Dengue Viruses Imported into Queensland, Australia, 2002–2010

**DOI:** 10.3201/eid1811.120014

**Published:** 2012-11

**Authors:** David Warrilow, Judith A. Northill, Alyssa T. Pyke

**Affiliations:** Queensland Health Forensic and Scientific Services, Archerfield, Queensland, Australia

**Keywords:** dengue, dengue virus, phylogenetics, importation, outbreak, Queensland, Australia, viruses

## Abstract

To assess risk for importation of dengue virus (DENV) into Queensland, Australia, and sources of imported viruses, we sequenced the envelope region of DENV isolates from symptomatic patients with a history of travel during 2002–2010. The number of imported dengue cases greatly increased over the surveillance period, some of which were associated with domestic outbreaks. Patients reported traveling to (in order) Asia, Papua New Guinea, Pacific Island countries, and non–Asia-Pacific countries. By using phylogenetic methods, we assigned DENV isolates from returning residents and overseas visitors with viremia to a specific genotypic group. Genotypes circulating in Asia were extremely diverse. Genotyping and molecular clock analysis supported Asian origination of a strain that caused an outbreak of DENV-4 in Pacific Island countries during 2007–2009, and subsequently, in Innisfail, Australia, in 2009. Our findings indicate that Asia is a major source of DENVs that are imported into Australia, causing a risk for epidemics.

## Abstract 2

Molecular epidemiologic analysis shows that travelers returning from Asia are the greatest source of risk.

Queensland, a state located in the tropical and subtropical northeastern area of Australia, has a long history of dengue virus (DENV) activity. Dengue was present in the late 19th century (*1*) and, following a lull for most of the 20th century, dengue importation and epidemic transmission have been increasingly reported in the past 20 years (*2*,*3*). Epidemics of the disease have occurred historically in other states of Australia, but only in Queensland have epidemics been reported in recent times. These epidemics were caused by the distribution of the vector, *Aedes aegypti* mosquitoes*.* The species was once found in other Australian states, but its area of distribution has now contracted so that it lies almost exclusively within Queensland’s borders (*4*,*5*).

Despite repeated transmission events, dengue is not endemic to Queensland, and transmission requires a viremic traveler to import the virus to initiate epidemic spread (*6*). Rapid identification of cases and disease tracking, incorporating targeted vector surveillance, and control measures adopted rigorously to limit epidemic potential have been major factors in preventing local transmission and in reducing the cost of managing mosquito-borne disease (*7*).

With the apparent increasing frequency of dengue epidemics and imported cases, the disease has become a major public health issue. Exposure to multiple serotypes of DENV, of which there are 4 in total, may result in a higher probability of potentially life-threatening conditions such as dengue hemorrhagic fever and dengue shock syndrome, a potentially life-threatening condition (*8*). Perhaps not coincidentally, 2 fatal cases of dengue hemorrhagic fever were reported in 2004 in Queensland, and the serologic profile of the case-patients indicated secondary infection consistent with dengue shock syndrome (*9*). Of additional concern is the possibility that the virus may become endemic if case numbers were to rise to a point at which vector control measures became ineffectual at controlling virus spread.

Recent DENV infection is diagnosed by serologic testing, through virus isolation or by nucleic acid amplification by reverse transcription PCR (RT-PCR). The advantage of the latter is that sequencing of reaction products enables a definitive diagnosis of acute infection, identification of the virus serotype, and genotyping. As an adjunct to isolation techniques, sequencing and genotyping can provide valuable evidence of importation or can confirm local transmission and enable differentiation between multiple circulating strains and serotypes. Analysis of DENV sequence data facilitates rapid disease tracking and vector control. However, not all specimens are suitable for RT-PCR because infected persons usually exhibit a relatively short-lived viremia early in the febrile period (*10*). In addition, clinicians may find it difficult to obtain acute-phase samples, particularly if patients delay their initial consultation or are still in transit during their viremic phase.

As part of control measures by Queensland Public Health, we sequenced the envelope region of DENV isolates from symptomatic patients with a history of travel during 2002–2010. The proportion of the 4 DENV serotypes that were imported was determined, as well as the geographic origin of each serotype. Phylogenetic trees containing imported DENV viruses and others strains circulating throughout the world (from GenBank) were constructed by using a maximum likelihood model. From this analysis, we ascertained the likely geographic origin of imported viruses. This enabled us to assess the risk for importation of DENV from various sources by travelers entering Australia. 

## Materials and Methods

### Virus Samples

Serum samples from patients with suspected DENV infection were referred to the Public Health Virology Laboratory, Queensland Health Forensic and Scientific Services, following the directive of Queensland Public Health medical officers, or were obtained through the public or private laboratory network. Acute-phase specimens underwent RT-PCR and serologic testing, and those that successfully yielded an RT-PCR product (after specific DENV serotype amplication) were sequenced and genotyped by phylogenetic analyses to assist public health investigations. This work was approved by the Ethics Committee of Queensland Health Forensic and Scientific Services.

### Viral RNA Extraction and Nucleotide Sequencing

RNA was extracted from 200 µL of serum, either manually (QIAamp viral RNA extraction kit; QIAGEN, Hildren, Germany) or by using the EZ1 Virus Mini Kit and (QIAGEN) according to the manufacturer’s instructions. Amplification was performed for each DENV serotype by using the Superscript III/Platinum Taq High Fidelity One-Step RT-PCR System (Invitrogen, Carlsbad, CA, USA) with specific RT-PCR primers (Table 1). Nucleotide sequencing of the complete envelope gene region (DENV-1, DENV-2, and DENV-4: 1,485 bp; DENV-3: 1,479 bp) was performed by using the Big Dye Terminator v3.1 cycle sequencing kit (Applied Biosystems, Foster City, CA, USA). Sequence data obtained were deposited in GenBank (Table 2).

**Table 1:** Amplification oligonucleotide primers for DENV genotyping RT-PCRs, Queensland, Australia, 2001–2010*†

DENV assay	Forward primer	Reverse primer
DENV-1‡	5′^760^-AACGTGGATGTCCTCTGAAGG-^780^3′	5′^1600^-CGAGGTCCAAGGCAGTG-^1584^3′
	5′^1418^-GCAACCATAACACCTCAA-^1435^3′	5′^2600^-TGGCTGATCGAATTCCACAC-^2581^3′
DENV-2§	5′-^789^GAAACATGCCCAGAGAATTGAAACT-^813^3′	5′-^1920^CCCTTCATATTGTACTCTGATAACTATTGTTCC-^1888^3′
	5′-^1547^AAGCTTGGCTGGTGCACAGGCAATGGTT-^1574^3′	5′-^2537^GGGGATTCTGGTTGGAACTTGTATTGTTCTGTCC-^2504^3′
DENV-3¶	5′-^291^TGGCTAGATGGGGTACCTTC-^310^3′ or5′-^722^GCTCCCCATGTCGGCATGGGACTGG-^746^3′	5′-^1819^CATCCCTTTGAGTTTCAATTTGTCCAT-^1793^3′
	5′-^1685^CTAGGATCTCAAGAAGGAGCAATGCA-^1710^3′	5′-^2550^ATGGCTGTTGCCACTCTTTTGGGGGA-^2525^3′
DENV-4#	5′-^742^TGGGATTGGAAACAAGAGCTGAGACATGGATGTC-^775^3′	5′-^1838^CGTGTATGACATTCCCTTGATTCTCAATTTCTCCA-^1804^3′
	5′-^1569^CAATGGTTTTTGGACCTACCTCTACCATGG-^1598^3′	5′-^2539^GGGGACTCTGGTTGAAATTTGTACTGTTCTGTCCA-^2505^3′

*DENV, dengue virus; RT-PCR, reverse transcription PCR. †Numbering is based on DENV-1 strain DENV-1BR/90 (AF226685), DENV-2 strain New Guinea C (AF038403), DENV-3 strain Ba51 (AY858037), DENV-4 strain Dominica 1981 (AF326573). For each DENV serotype, forward primers were paired with the shown respective reverse primers in two separate genotyping RT-PCR assays (A. Pyke, unpub. method). ‡A. Pyke and D. Beasley, unpub. method. §S. Mei Lok, unpub. data. ¶I. Serafin and A. Pyke, unpub. method. #C. Howard and A. Pyke, unpub. method.

**Table 2:** Description of DENV virus strains analyzed, Queensland, Australia, 2002–2010*

Serotype/sequence name	Geographic origin	Year isolated	GenBank accession no.
DENV-1			
Bali 2003	Bali	2003	JN415488
Bali 2010a	Bali	2010	JN415489
Bali 2010b	Bali	2010	JN415490
Bali 2010c	Bali	2010	JN415491
Bali 2010d	Bali	2010	JN415492
Bali 2010e	Bali	2010	JN415493
Bali 2010f	Bali	2010	JN415494
Cairns 2003	Cairns, Australia	2003	JN415495
Cambodia 2007	Cambodia	2007	JN415496
Cook Islands 2002	Cook Islands	2002	JN415497
Cook Islands 2006	Cook Islands	2006	JN415498
East Timor 2000	Timor-Leste	2000	JN415499
East Timor 2008	Timor-Leste	2008	JN415500
East Timor 2009	Timor-Leste	2009	JN415501
East Timor 2010	Timor-Leste	2010	JN415502
Fiji 2002	Fiji	2002	JN415503
Fiji 2006a	Fiji	2006	JN415504
Fiji 2006b	Fiji	2006	JN415505
Guyana 2008	Guyana	2008	JN415506
India 2008	India	2008	JN415507
India 2010	India	2010	JN415486
Indonesia 2010a	Indonesia	2010	JN415508
Indonesia 2010b	Indonesia	2010	JN415510
Jakarta 2004	Jakarta	2004	AY858983†
Laos 2007	Laos	2007	JN415509
Malaysia 1972	Malaysia	1972	AF425622†
Malaysia 2005	Malaysia	2005	JN415511
Malaysia 2008	Malaysia	2008	JN415512
Malaysia 2010	Malaysia	2010	JN415513
Mareeba 2003	Mareeba, Australia	2003	JN415514
Palau 2000	Palau	2000	JN415515
Philippines 2005	The Philippines	2005	JN415516
Philippines 2010	The Philippines	2010	JN415517
PNG 2003	Papua New Guinea	2003	JN415518
PNG 2009	Papua New Guinea	2009	JN415519
Samoa 2001	Samoa	2001	JN415520
Singapore 2003	Singapore	2003	FJ469907†
Singapore 2005	Singapore	2005	EU081246†
Singapore 2005	Singapore	2005	EU081247†
Singapore 2008	Singapore	2008	JN415521
Solomon Islands 2002	Solomon Islands	2002	JN415522
Southeast Asia 2007	Southeast Asia	2007	JN415523
Southeast Asia 2005	Southeast Asia	2005	JN415529
Sri Lanka 2004	Sri Lanka	2004	JN415524
Sumatra 1998	Sumatra	1998	AB189121†
Thailand 1954	Thailand	1954	D10513†
Thailand 1980	Thailand	1980	AY732474†
Thailand 2001	Thailand	2001	JN415525
Thailand 2008a	Thailand	2008	JN415526
Thailand 2008b	Thailand	2008	JN415527
Thailand 2010	Thailand	2010	JN415528
Tonga 2008	Tonga	2008	JN415530
Townsville 2008	Townsville, Australia	2008	JN415531
Townsville 2009	Townsville, Australia	2009	JN415532
Venezuela 2007	Venezuela	2007	EU482609†
Vietnam 2006	Vietnam	2006	JN415533
Vietnam 2006	Vietnam	2006	EU482818†
Vietnam 2008a	Vietnam	2008	JN415534
Vietnam 2008b	Vietnam	2008	JN415535
Vietnam South 2008	Vietnam	2008	GU131812†
Vietnam 2010	Vietnam	2010	JN415487
Yap Island 2004	Yap Island	2004	AB204803†
DENV-2			
Bali 2009	Bali	2009	JN568242
Bali 2010	Bali	2010	JN568243
Borneo 2009	Borneo	2009	JN568247
Brunei 2005	Brunei	2005	EU179858†
Cairns 2003a	Cairns, Australia	2003	JN568248
Cairns 2003b	Cairns, Australia	2003	JN568249
Cairns 2004	Cairns, Australia	2004	JN568250
Cairns 2006	Cairns, Australia	2006	JN568251
Cairns 2008	Cairns, Australia	2008	JN568252
Cairns 2010	Cairns, Australia	2010	JN568253
Cambodia 2003	Cambodia	2003	GQ868621†
Cambodia 2008	Cambodia	2008	GU131924†
China 2001	China	2001	EF051521†
East Timor 2000	Timor-Leste	2000	JN568254
East Timor 2002	Timor-Leste	2002	JN568255
East Timor 2004	Timor-Leste	2004	JN568256
East Timor 2010	Timor-Leste	2010	JN568257
India 2001	India	2001	DQ448237†
India 2009	India	2009	JN568258
India 2003	India	2003	JN568260
India 2010	India	2010	JN568259
Indonesia 2004	Indonesia	2004	AY858035†
Kuranda 2002	Kuranda, Australia	2002	JN568261
Laos 2010	Laos	2010	JN568244
Mt Isa 2010	Mt Isa, Australia	2010	JN568262
New Guinea C 1944	New Guinea	1944	AF038403†
Peru 1996	Peru	1996	IQT1797†
Philippines 2003	The Philippines	2003	JN568263
Philippines 2010a	The Philippines	2010	JN568264
Philippines 2010b	The Philippines	2010	JN568265
PNG 2003	PNG	2003	JN568266
PNG 2009	PNG	2009	JN568241
PNG 2010a	PNG	2010	JN568267
PNG 2010b	PNG	2010	JN568268
PNG 2010c	PNG	2010	JN568269
PNG 2010d	PNG	2010	JN568270
Singapore 2008	Singapore	2008	GU370051†
Southeast Asia 2010b	Southeast Asia	2010	JN568276
Southeast Asia 2010a	Southeast Asia	2010	JN568277
Sri Lanka 1996	Sri Lanka	1996	FJ882602†
Sumatra 2009	Sumatra	2009	JN568271
Sumatra 2010	Sumatra	2010	JN568272
Taiwan 2001	Taiwan	2001	DQ645541†
Thailand 1996	Thailand	1996	AF100459†
Thailand 2001	Thailand	2001	DQ181797†
Thailand 2007	Thailand	2007	JN568273
Thailand 2010a	Thailand	2010	JN568274
Thailand 2010b	Thailand	2010	JN568275
Thailand 2010c	Thailand	2010	JN568245
Torres Strait 2003	Torres Strait, Australia	2003	JN568278
Townsville 1993	Townsville, Australia	1993	AY037116†
Townsville 2010a	Townsville, Australia	2010	JN568279
Townsville 2010b	Townsville, Australia	2010	JN568246
Tully 2010	Tully, Australia	2010	JN568280
Venezuela 1990	Venezuela	1990	GQ868540†
Vietnam 2005	Vietnam	2005	FM210207†
Vietnam 2006	Vietnam	2006	EU569721†
Vietnam 2010a	Vietnam	2010	JN568281
Vietnam 2010b	Vietnam	2010	JN568282
DENV-3			
Bali 2009	Bali	2009	JN568284
Bali 2010a	Bali	2010	JN568283
Bali 2010b	Bali	2010	JN575560
Bali 2010c	Bali	2010	JN575561
Cairns 1998	Cairns, Australia	1998	JN575562
Cairns 2008a	Cairns, Australia	2008	JN575563
Cairns 2008	Cairns, Australia	2008	JN575564
Cambodia 2006	Cambodia	2006	JN575565
East Timor 2000	Timor-Leste	2000	JN575566
Fiji 1992	Fiji	1992	L11422†
India 1984	India	1984	L11424†
Indonesia 1985	Indonesia	1985	L11428†
Indonesia 1998	Indonesia	1998	AY265857†
Indonesia 2004a	Indonesia	2004	AY858037†
Indonesia 2004b	Indonesia	2004	AY858047†
Indonesia 2008a	Indonesia	2008	JN575567
Indonesia 2008b	Indonesia	2008	JN575568
Philippines 1983	The Philippines	1983	L11432†
Philippines 1997	The Philippines	1997	AY496879†
Philippines 2010	The Philippines	2010	JN575570
PNG 2008	Papua New Guinea	2008	JN575571
PNG 2010a	Papua New Guinea	2010	JN575572
PNG 2010b	Papua New Guinea	2010	JN575573
Puerto Rico 1977	Puerto Rico	1977	L11434†
Samoa 1986	Samoa	1986	L11435†
Singapore 2005	Singapore	2005	EU081221†
Southeast Asia 2008	Southeast Asia	2008	JN575569
Sri Lanka 1991	Sri Lanka	1991	L11438†
Tahiti 1989	Tahiti	1989	L11619†
Taiwan 1998	Taiwan	1998	DQ675532†
Taiwan 1999	Taiwan	1999	DQ675533†
Thailand 1973	Thailand	1973	L11620†
Thailand 1987	Thailand	1987	L11442†
Thailand 1997a	Thailand	1997	JN575574
Thailand 1997b	Thailand	1997	JN575575
Thailand 2010	Thailand	2010	JN575576
Townsville 2006	Townsville, Australia	2006	JN575577
Townsville 2007	Townsville, Australia	2007	JN575578
Townsville 2009	Townsville, Australia	2009	JN575579
Vietnam 2007	Vietnam	2007	EU482461†
Vietnam 2008	Vietnam	2008	JN575580
DENV-4			
Bali 2010	Bali	2010	JN575583
Brazil 1982	Brazil	1982	U18425†
Cairns 2002	Cairns, Australia	2002	JN575584
China 2001	China	2001	AF289029†
Cook Islands 2009	Cook Islands	2009	JN575582
Dominica 1981	Dominica	1981	AF326573†
East Timor 2000	Timor-Leste	2000	JN575585
East Timor 2007	Timor-Leste	2007	JN575586
El Salvador 1983	El Salvador	1983	U18426†
Fiji 2008	Fiji	2008	JN575587
Indonesia 1973	Indonesia	1973	U18428†
Indonesia 1977	Indonesia	1977	U18430†
Indonesia 2010a	Indonesia	2010	JN575588
Indonesia 2010b	Indonesia	2010	JN575589
Innisfail 2009	Innisfail, Australia	2009	JN575581
Jakarta 2004	Jakarta	2004	AY858049†
Malaysia 2009	Malaysia	2009	JN575590
New Caledonia 1984	New Caledonia	1984	U18432†
Philippines 1984	The Philippines	1984	U18435†
Philippines 2004	The Philippines	2004	JN575591
Puerto Rico 1986	Puerto Rico	1986	U18436†
Samoa 2008	Samoa	2008	JN575592
Solomon Islands 2008	Solomon Islands	2008	JN575593
Sri Lanka 1978	Sri Lanka	1978	U18437†
Tahiti 1985	Tahiti	1985	U18439†
Thailand 1978	Thailand	1978	U18441†
Thailand 1984	Thailand	1984	U18442†
Thailand 1997	Thailand	1997	AY618988†
Thailand 2001	Thailand	2001	AY618992†
Thailand 2010	Thailand	2010	JN575594
Torres Strait 2005	Torres Strait, Australia	2005	JN575595
Townsville 2005	Townsville, Australia	2005	JN575596

*DENV, dengue virus; PNG, Papua New Guinea. †Sequences obtained from GenBank.

### Phylogenetic Analysis of Envelope Protein Sequence

Full-envelope protein sequences for each serotype were aligned by using the multiple alignment tool of MEGA5 (www.megasoftware.net). Unrooted trees were then constructed by using a maximum likelihood estimation with a Jukes-Cantor model and γ-distributed rates, and by constructing 1,000 replicates to generate bootstrap support values. Divergence time from a common ancestor was estimated by using the molecular clock calculator.

## Results

### Increasing Incidence of Dengue Outbreaks and Serotype Diversity

**Figure 1:**
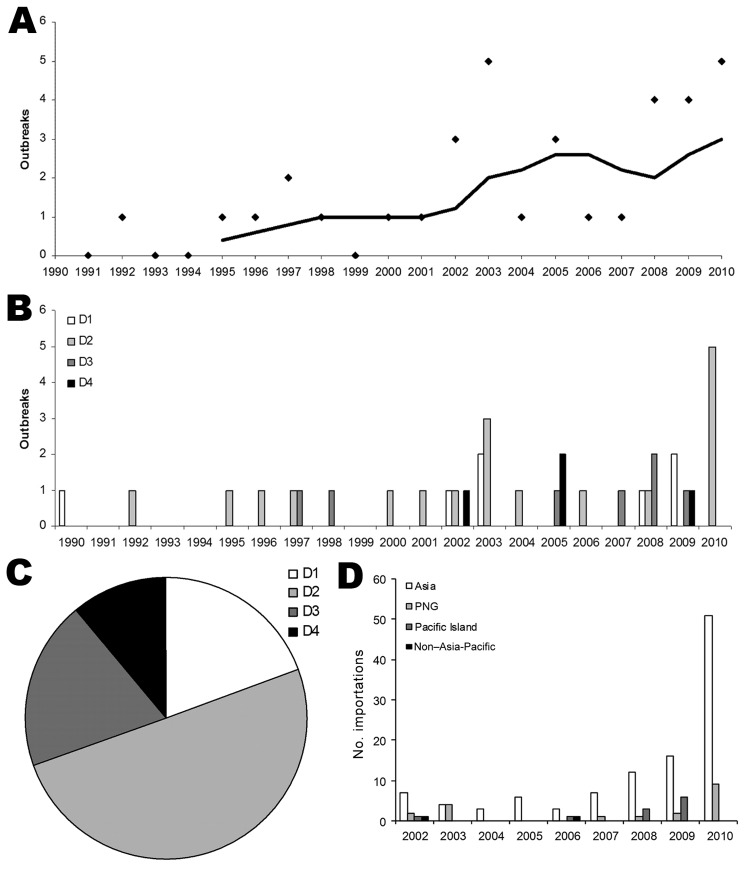
Number and diversity of dengue outbreaks in northern Queensland, Australia. A) Outbreaks of dengue causing epidemic spread in Queensland 1990–2010 showing 5-year moving average. B) Outbreaks shown as individual serotypes. C) Proportion of dengue virus serotypes responsible for the outbreaks shown in A and B. D) Geographic origins of dengue viruses imported into Queensland by viremic travelers. D1–D4, DENV-1–DENV-4; PNG, Papua New Guinea.

Previous reports (*3*,*11*) and anecdotal evidence indicated that there has been an increase in the number of dengue outbreaks occurring in Queensland. To investigate this apparent trend, we combined recent and historical outbreak data (*3*) over a 20-year period. A 5-year moving average does indeed show trends of increasing dengue outbreak incidence and increasing diversity of DENV serotypes that cause such outbreaks (Figure 1, panels A, B). A line of best fit revealed a significant increase with time (r^2^ = 0.48; p<0.05 by Student *t* test, 2-tailed). All 4 DENV serotypes caused outbreaks; DENV-2 was the most common cause (50.0%), followed by DENV1 and DENV-3 (19.4% each) and DENV-4 (11.1%) (Figure 1, panel C). The increase in outbreak incidence reflects changes in international travel over this period, which has increased 3.5-fold since the early 1990s (*12*). This increase is consistent with increased importations of virus carried by viremic travelers and the recognized increase in DENV infections throughout the world (*13*). Also of note is the dramatic increase in infections caused by imported viruses in 2010 (Figure 1, panel D).

### Geographic Origins and Diversity of Imported DENVs, 2002–2010

We ascertained the number and diversity of imported DENV serotypes from infected travelers during 2002–2010 (Table 3). This period was chosen because the most comprehensive patient sequence data were available. Information was analyzed from viremic travelers, for whom an RT-PCR amplification product and serotype designation could be obtained. The possible strain origins, which were determined after phylogenetic analyses, were compared with available travel histories to ascertain likely geographic origins of the infecting virus. The data were categorized into 4 separate regions: Asia, Papua New Guinea (PNG), the Pacific Islands, and countries outside of the Asia-Pacific region (non–Asia-Pacific).

**Table 3:** Number and diversity of imported dengue serotypes, Queensland, Australia, 2002–2010*

Region and country	No. (%) cases	Dengue serotypes (genotypes)
Asia		
Indonesia	37 (26.4)	1 (I, IV), 2 (Cosmopolitan), 3 (I), 4 (II)
Thailand	15 (10.7)	1 (I), 2 (Asian genotype I), 3 (II), 4 (I)
Philippines	10 (7.1)	1 (IV), 2 (Cosmopolitan), 3 (I), 4 (I)
India	9 (6.4)	1 (V), 2 (Cosmopolitan)
Timor-Leste	9 (6.4)	1 (IV), 2 (Cosmopolitan), 4 (II)
Vietnam	7 (5.0)	1 (I), 2 (Asian genotype I), 3 (II)
Malaysia	5 (3.6)	1 (I, IV), 4 (II)
Laos	2 (1.4)	1 (I), 2 (Asian genotype I)
Cambodia	2 (1.4)	1 (I), 3 (II)
Singapore	1 (0.7)	1 (I)
Sri Lanka	1 (0.7)	1 (V)
Asia, not specified	11 (7.9)	-
Papua New Guinea	19 (13.6)	1 (I, IV), 2 (Cosmopolitan), 3 (I)
Pacific Islands		
Fiji	4 (2.9)	1 (IV), 4 (II)
Samoa	2 (1.4)	4 (II)
Solomon Islands	1 (0.7)	1 (ND)
Tonga	3 (2.1)	4 (ND)
Vanuatu	1 (0.7)	4 (ND)
Non–Asia-Pacific		
Brazil	1 (0.7)	3 (ND)
Guyana	1 (0.7)	1 (V)
Total	140	

*ND, not determined

Most infected travelers (77.9%) reported spending time abroad in Asia. In particular, 26.4% of all virus importations could be traced to Indonesia alone. All 4 DENV serotypes were detected in the specimens sequenced from persons with a travel history to that country. Notably, patients reported traveling to other Asian countries, including Thailand and the Philippines, where all 4 DENV serotypes have been found. Most other countries to which travel was reported had at least 3 DENV serotypes (Timor-Leste, PNG, and Vietnam), 2 serotypes (Cambodia, India, Fiji, Malaysia, and Laos), or 1 serotype (Brazil, Guyana, Samoa, Singapore, Solomon Islands, Sri Lanka, Tonga, and Vanuatu). A greater degree of diversity cannot be excluded in many of these countries because sampling numbers for individual countries were often low.

**Figure 2:**
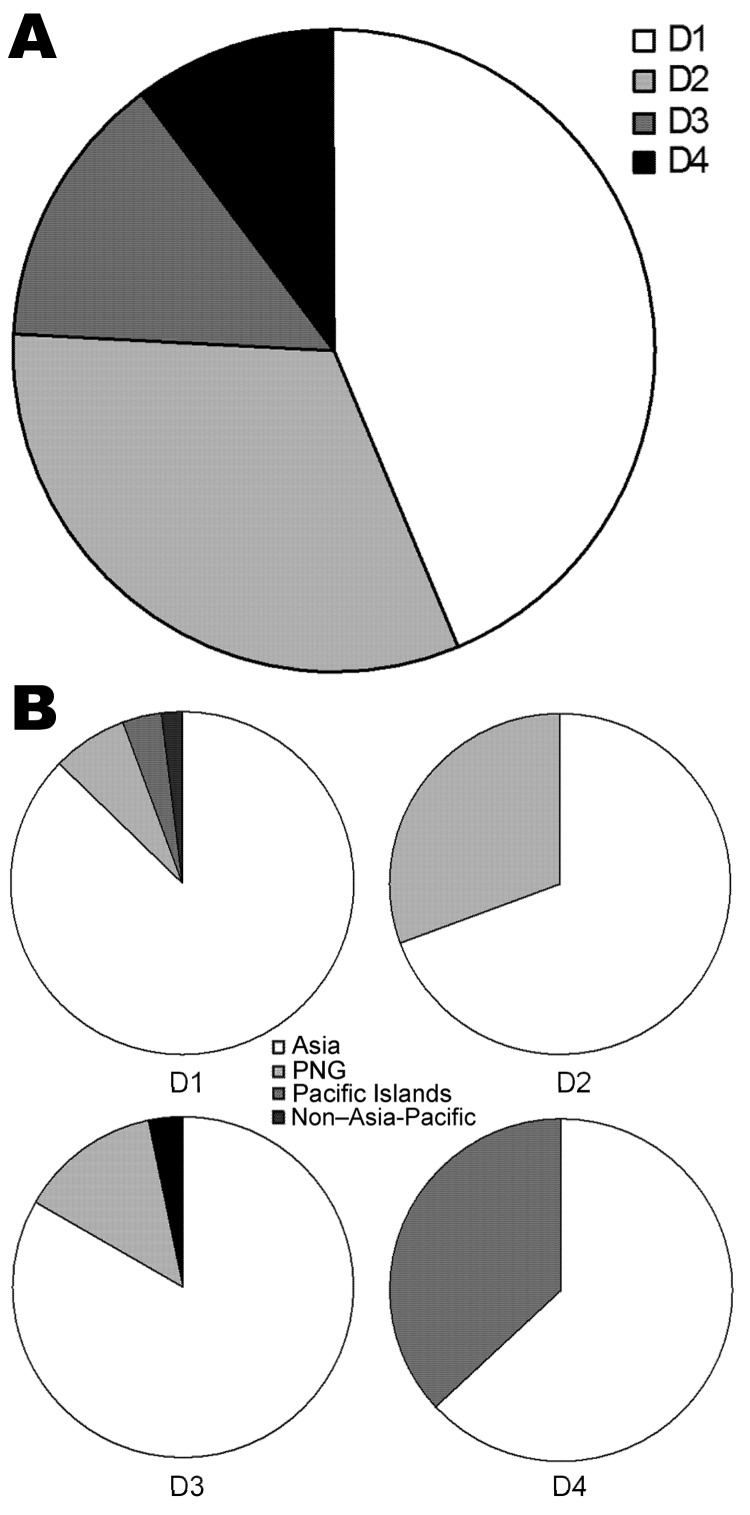
Importation of dengue viruses (DENVs) into Queensland, Australia, 2002–2010. A) Proportion of imported DENV serotypes. B) Geographic origins of the 4 imported DENV serotypes. D1–D4, DENV-1–DENV-4; PNG, Papua New Guinea.

We calculated the proportion of the 4 DENV serotypes for infected travelers (Figure 2, panel A), which was slightly different from the proportion associated with outbreaks within Queensland (Figure 1, panel C). The serotype most commonly imported by travelers was DENV-1 (39.3%), followed by DENV-2 (25.7%), DENV-3 (21.4%), and DENV-4 (13.6%). Strains of all 4 DENV serotypes originated mainly in Asia (Figure 2, panel B). DENV-1 had the most diverse origins, with patients reporting travel mainly to Asia, but also to PNG, the Pacific Islands, and non–Asia-Pacific regions. In addition to Asia, DENV-2 was found to originate in PNG; DENV-3 originated in PNG and a non–Asia-Pacific area (Brazil); and DENV-4 originated in the Pacific Islands.

### Origins of DENVs Imported by Returning Residents

Infected travelers were either returning Queensland residents or international visitors. Returning residents were the largest proportion of patients (96.4%) who sought treatment from the health system with dengue viremia. Further analysis was conducted on the subset of returning residents (135 of 140 patients with imported cases). Similarly to the analysis of all travelers above, infected returning residents (Table 4) reported that they had most frequently returned from Asia (77.0%), followed by PNG (13.3%), then the Pacific Islands (8.1%), and least often from non–Asia-Pacific areas (1.5%). The overall ratio was significantly different from that expected on the bases of the proportion of all Queensland residents who reported returning from dengue-endemic countries of those 4 regions, as calculated by using data from the Australian Bureau of Statistics for 2002–2010 (χ^2^ analysis, p = 0.0004). The proportion of patients reporting travel to Asia, and to PNG in particular, was higher than expected. The overrepresentation of cases from PNG is best explained by a recent increase in DENV activity in that country, which is consistent with a large number of importations from PNG (47.4%) during 2010 (Figure 1, panel D) and, in addition, other recent reports (*14*). In comparison, the proportion of patients who reported travel to the Pacific Islands was lower than expected. Because only 2 cases originated in the non–Asia-Pacific region, it was subsequently difficult to draw conclusions about this region for statistical purposes.

**Table 4:** Observed and expected numbers of imported DENVs by region, Queensland, Australia, 2002–2010*

Imported DENVs	No. (%) from Asia†	No. (%) from PNG	No. (%) from Pacific Islands	No. (%) from non–Asia-Pacific region
Observed	104 (77.0)	18 (13.3)	11 (8.1)	2 (1.5)
Expected‡	92 (68.2)	11 (7.9)	28 (20.8)	4 (3.2)

*χ^2^ is 18 (p<0.0004; 2-tailed test); DENVs, dengue viruses; PNG, Papua New Guinea. †Asia includes travelers to Indonesia, Timor-Leste, Thailand, India, Malaysia, Philippines, Vietnam, Singapore, Cambodia, Sri Lanka and Laos; Papua New Guinea; Pacific Islands includes travelers to Fiji, Samoa, Solomon Islands, Tonga, and Vanuatu; non–Asia-Pacific region was defined as all cases outside the Asia-Pacific region which included Brazil and Guyana. ‡Based on travel data from the Australian Bureau of Statistics (www.abs.gov.au/) for departing Queensland residents who named the country where they planned to spend the most time, selected for those countries designated as having an ongoing dengue transmission risk, according to the Centers for Disease Control and Prevention Dengue Map (www.healthmap.org/dengue).

### Genotype Assignment of Imported DENVs

**Figure 3:**
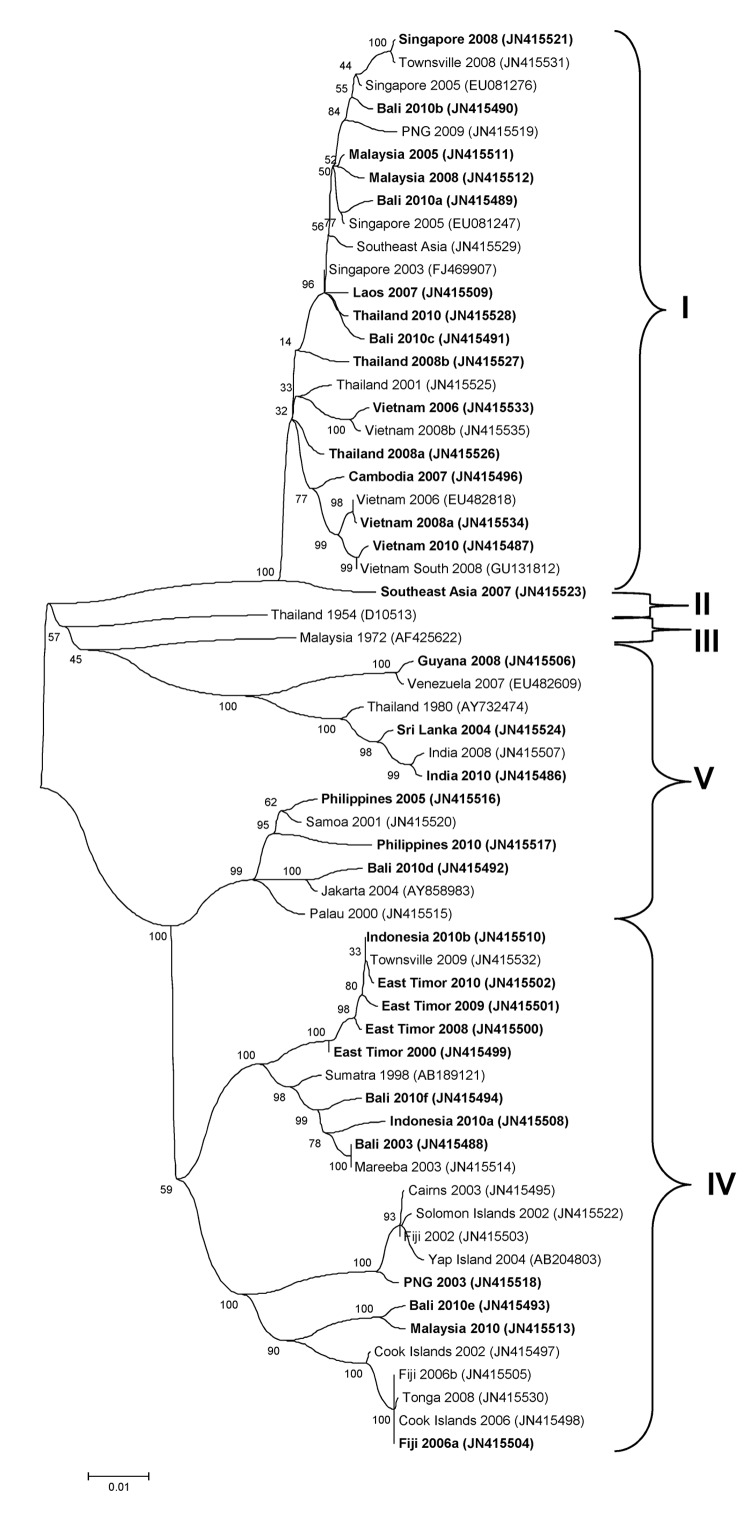
Phylogenetic tree showing the relationship of dengue viruses, serotype 1, imported into Queensland, Australia, 2001–2010, based on sequencing of the envelope gene. Viruses are designated according to reported origin and GenBank accession number, and imported cases are shown in **boldface**. Genotypes are indicated on the right. Scale bar indicates nucleotide substitutions per site.

**Figure 4:**
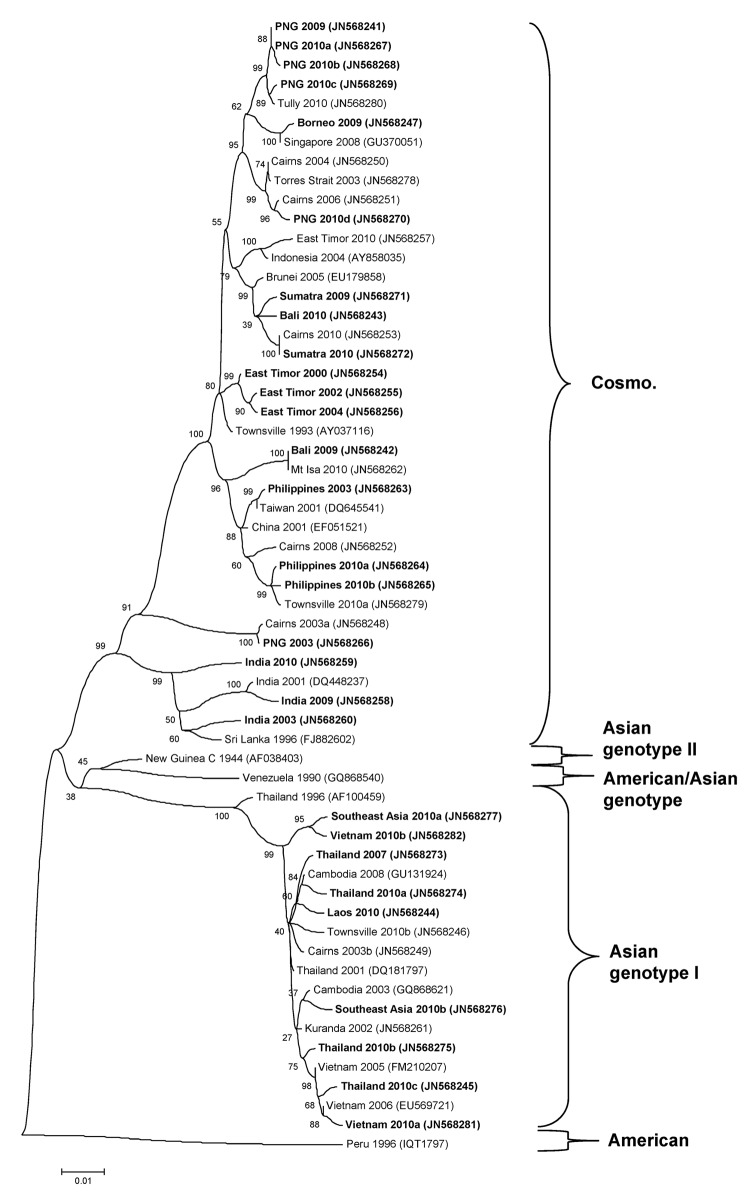
Phylogenetic tree showing the relationship of dengue viruses, serotype 2, that were imported into Queensland, Australia, 2002–2010, based on sequencing of the envelope gene. Viruses are designated according to reported origin and GenBank accession number, and imported cases are shown in **boldface**. Genotypes are indicated on the right. Cosmo, Cosmopolitan. Scale bar indicates nucleotide substitutions per site.

**Figure 5:**
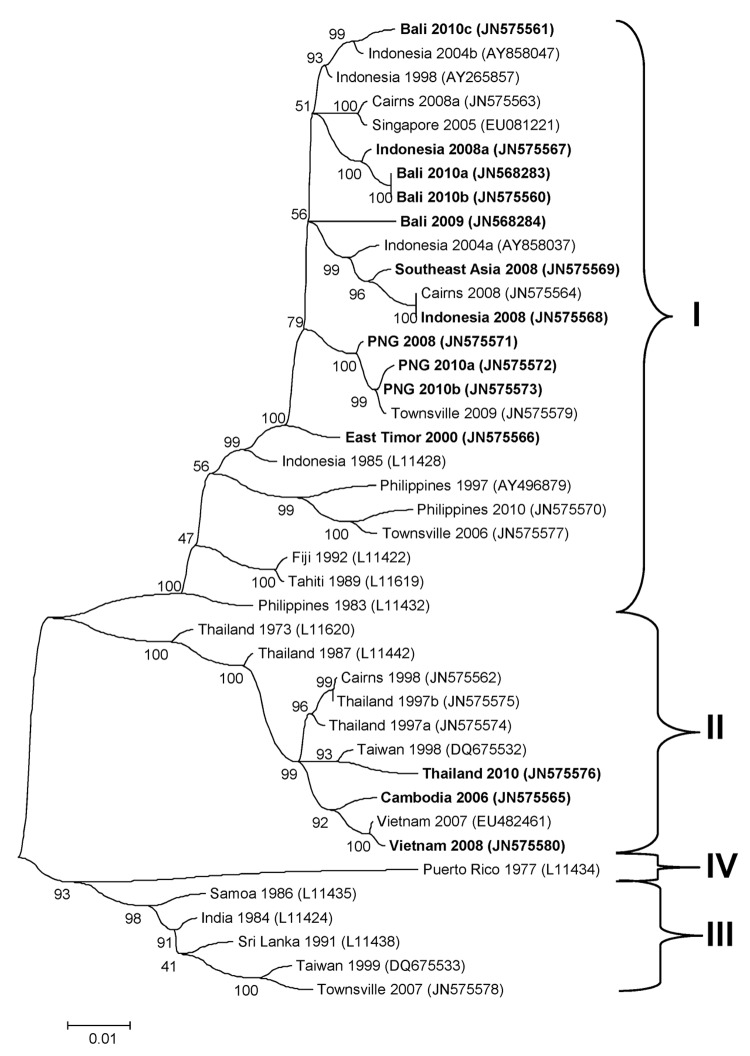
Phylogenetic tree showing the relationship of dengue viruses, serotype 3, imported into Queensland, Australia, 2002–2010, based on sequencing of the envelope gene. Viruses are designated according to reported origin and GenBank accession number, and imported cases are shown in **boldface**. Genotypes are indicated on the right. Scale bar indicates nucleotide substitutions per site.

**Figure 6:**
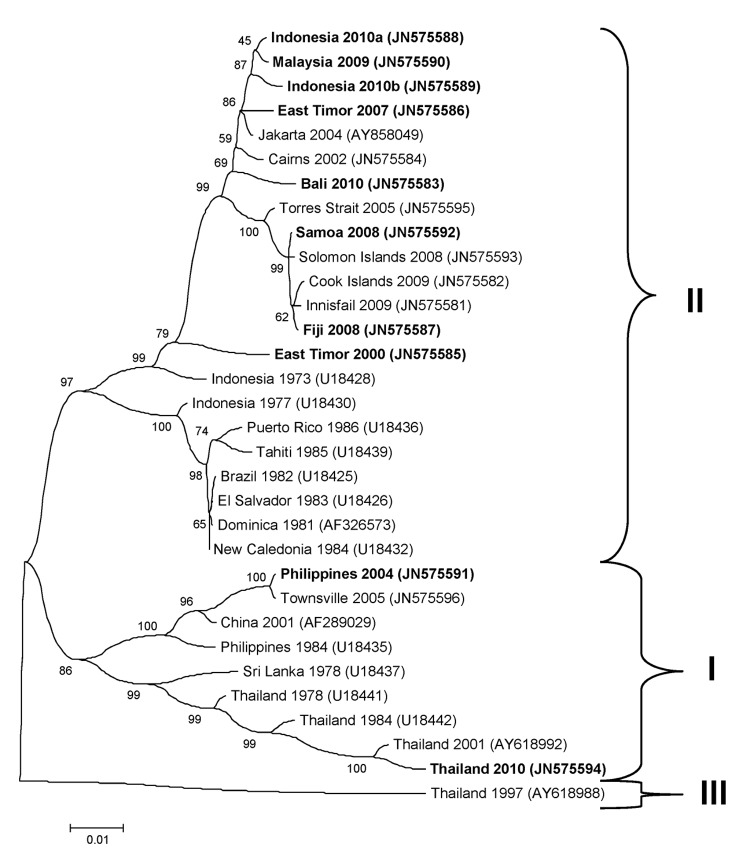
Phylogenetic tree showing the relationship of dengue viruses, serotype 4, imported into Queensland, Australia, 2002–2010, based on sequencing of the envelope gene. Viruses are designated according to reported origin and GenBank accession number, and imported cases are shown in **boldface**. Genotypes are indicated on the right. Scale bar indicates nucleotide substitutions per site.

The genotypic mix for the various regions from which dengue was imported is shown in Table 3 and Figure 3, Figure 4, Figure 5, Figure 6. Across all regions, viruses could be classified into 1 of 2 genotypic groups within each serotype; the exception was DENV-1, which had 3 groups. In countries which were a source of imported viruses, generally 1 genotypic group for each serotype predominated.

DENV genotypic groups generally circulate in particular regions (*15*). The viruses imported into Queensland were consistent with DENV genotypes which had previously been reported to circulate in those countries to which patients had reported travel (*16*–*19*). For example, DENV-4 genotypic group II has been reported in Indonesia, Tahiti, the Caribbean Islands, and Central and South America (*17*). In 2007–9, DENV-4 was introduced into the Pacific Islands, displacing DENV-1 in the process (*20*,*21*). Our genotypic analysis confirms classification of the Pacific Island DENV-4 in genoptypic group II, as recently reported (*21*). This was the first time this genotypic group had been reported in the Pacific region, and suggested that the origin of this strain of DENV-4 may have been Southeast Asia.

In support of this suggestion, a closely related DENV-4 strain from the Torres Strait (Figure 6, JN575595) with 99.1% envelope nucleotide identity to a DENV-4 strain from Samoa (Figure 6, JN575592), was detected before the Pacific outbreak in 2005. A maximum likelihood test of the phylogenetic tree determined that a molecular clock was applicable (H_o_ not rejected; p = 0.06). Using a previously published substitution rate for dengue 4 of 1 × 10^−3^ substitutions/site/year (*22*), we calculated that divergence from a common ancestor occurred in ≈2002 with an error of ± 2 years. Thus, the Pacific Island outbreak strain (Figure 6, Pacific Island clade) is geographically and temporally closely related to the Torres Strait 2005 virus. Both of these virus strains are mostly closely related to DENV-4 strains which originated in Indonesia (Figure 6, JN575583). These data support suggestions the Pacific Island outbreak strain originated in Indonesia and made its way to the Torres Strait (Australia) in 2005, probably through PNG, and into the Pacific in 2007 where it is currently circulating. A virus most closely related to the Pacific Island strain was then imported into Innisfail in northern Queensland in 2009, where it caused an outbreak (Figure 6). This incident highlights the epidemic potential of DENV strains that are imported into Queensland (*3*,*23*,*24*).

## Discussion

In this study we have analyzed the importation of DENVs into northern Queensland, the only area within Australia where domestic epidemic spread is a risk. Two issues are apparent from these analyses. First, DENV infections, in terms of the number of importations and outbreaks, have increased in recent years. This issue is most apparent when it is considered that 42.9% of all instances of virus importation identified in this study occurred in 2010. The greatest risk was from residents returning from travel overseas, rather than overseas visitors. However, cases in the latter may be somewhat underreported because they may be more reluctant to seek medical assistance in a foreign country.

The second issue is the large degree of risk that Asia represents as a primary source of DENVs that can cause epidemics in Australia. Not only does Asia represent the biggest source of imported viruses in terms of number and serotype diversity, but it is also a source of viruses that can be imported into the Pacific region and, subsequently, a secondary source of importation into Australia as can be seen from the outbreak of the Pacific Island DENV-4 genotypic group II in Innisfail in 2009. If suggestions that the Pacific Island states are unable to sustain long-term DENV circulation are correct (*20*), then Asia may also be an important source of new outbreaks in the Pacific by incursions perhaps from either PNG or the islands of the Torres Strait.

To determine whether travelers returning from the 4 regions were either underrepresented or overrepresented in the dataset, we compared travelers departing Australia (using information obtained from outgoing passenger cards). A subset of Queensland residents was used as this information was available from the Australian Bureau of Statistics only for outgoing residents. The overrepresentation of infections imported from Asia and PNG relative to the Pacific Island countries may be due to higher levels of DENV activity in those countries. In the case of PNG, this result was mostly due to a large increase in imported DENVs from that country in 2010 (47.4% of all imported DENVs from PNG). Data from 2011 continue this trend to higher levels of DENVs imported from that country (data not shown). A previous study noted a decline in imported DENVS from PNG from 51% over the period 1999–2003 to 12% from 2004 to 2008 (*25*). The findings from this study may indicate a return to the historically higher proportion of imported DENVs from that region with the likelihood that recent dengue activity in PNG has intensified.

Little is known about overt disease in adults who acquire DENV-2 and DENV-4 infections. Disease may only be seen in those persons with previous antibody responses to another dengue serotype (*26*). This circumstance has implications for vaccine development because those persons with DENV antibodies may experience disease when exposed to vaccine formulations that contain apparently attenuated DENV-2 and DENV-4 (*26*). Susceptible adults who contract dengue while traveling represent an opportunity to study the factors associated with overt disease. To explore the pathogenicity of DENV-2 and DENV-4, serologic responses should be correlated, in the context of patient age, with molecular diagnostics in future studies of dengue surveillance.

This work clearly shows the increasing risk that viremic travelers pose to Australia, and to Queensland in particular, as a means for importing DENVs that could have substantial outbreak potential. Molecular epidemiologic studies have identified Asia as the greatest source of DENV infections that have been imported into Queensland recently. The increase in imported DENV strains and the number of outbreaks is of major public health importance and has been largely exacerbated by the heightened frequency and affordability of modern air travel. As this trend continues, the chance of the virus becoming endemic and the likelihood of the recurrence of disease also increase. Although additional studies are required to investigate the clinical implications of the imported viruses and specific patient anomalies, the sequence information presented here could assist future understanding of viral markers in relation to symptomatic disease and their association with pathogenesis.
